# Evaluation of *Aloe weloensis* (Aloeacea) Mucilages as a Pharmaceutical Suspending Agent

**DOI:** 10.1155/2021/6634275

**Published:** 2021-05-19

**Authors:** Yohannes Mengesha, Abdu Tuha, Yimer Seid, Admassu Assen Adem

**Affiliations:** Department of Pharmacy, College of Medicine and Health Sciences, Wollo University, P.O. Box 1145, Dessie, Ethiopia

## Abstract

Natural polymers, specifically mucilages, have been used as a suspending agent for a long period of time. Natural excipients can serve as an alternative to synthetic products since they are less expensive, less toxic, and devoid of environmental pollution. There are many species of *Aloe* found in Ethiopia which can be used as a source of mucilage. In this study, mucilage from *Aloe weloensis,* which is found in Wollo floristic region, was extracted and tested as a suspending agent at different suspending agent concentrations and compared with standard suspending agents (acacia and sodium carboxy methylcellulose (NaCMC)) by formulating zinc oxide suspension. The mucilage obtained from *Aloe weloensis* leaves has shown comparable suspending agent ability with acacia. The rate of sedimentation and viscosity was higher at 1% and 4% mucilage concentrations than acacia though the difference was not significant (*p* > 0.05). The suspension was slightly basic and easily dispersible than NaCMC. Suspensions formulated from NaCMC were superior in terms of viscosity and sedimentation volume which was significantly different (*p* < 0.05) accompanied by lower flow rates than suspensions formulated from acacia and *Aloe weloensis* mucilages. The results suggested that *Aloe weloensis* mucilage could be used as an alternative suspending agent.

## 1. Introduction

Pharmaceutical suspensions are biphasic and thermodynamically unstable and consist of a dispersion containing finely divided insoluble material suspended in a liquid medium [1]. To obtain a pharmaceutically acceptable, thermodynamically stable suspension, there is a need to include a suspending agent in a dosage form. It maintains a uniform dispersion of particles that would otherwise settle rapidly to form closely packed sediment and prevent the removal of an accurate dose both by forming a physical barrier to aggregation and increasing the consistency of the suspending medium [2].

Among the commonly used suspending agents are natural polysaccharides (acacia, guar gum, agar, xanthan gum, and tragacanth), semisynthetic polysaccharides (sodium carboxy methylcellulose), clays, and synthetic agents [1]. In recent times, focus on plant research has increased all over the world, and a large body of evidence has been collected to show the immense potential of plants used in various pharmaceutical applications [3]. And hence, new and improved excipients continue to be developed for conventional drug delivery systems and also to meet the needs of modern and better formulation [4].

Plant mucilages are preferred to semisynthetic and synthetic excipients because they are biocompatible, biodegradable, cheap and easily available, nontoxic, nonirritant, and amenable to both chemical and biochemical modifications. These characteristics make them a very ideal candidate to be used in pharmaceutical formulations [3]. Studies have shown that mucilages from different parts of a plant have been tested as a suspending agent such as from the roots of *Cissus vitiginea* [5] and *Curculigo orchioides* [1] and from leaves of *S. oleracea L*. [6] and gave promising results to be used as a pharmaceutical suspending agent. *Aloe* mucilage from other species have been tested as pharmaceutical excipients such as suspending agent [2], gelling agent [7] tablet binder [8], and tablet sustain releasing agent [9]. Mucilages from chia seeds [10] and okra fruit [11] have been tested as a binder. Mucilage from hibiscus leaves has shown promising result as a suspending agent [12].

In our county, Ethiopia there are plenty of plants which can be used as a source of many pharmaceutical excipients including 38 species of *Aloe*. *Aloe* has many species found in Ethiopia among them *Aloe* found in Wollo floristic region is one of the potential source of mucilage [13]. *Aloe weloensis*, locally known as Eret tafa, belongs to the family Aloaceae [14], and its mucilage has never been tested as a suspending agent. However, the antibacterial and antimalarial activity of the plant has been tested [14, 15]. Hence, it is wise to search for new suspending agents from the mucilage of *Aloe weloensis.*

## 2. Methods and Materials

### 2.1. Materials

Fresh leaves of *Aloe weloensis* was harvested from Dessie, northeastern Ethiopia. The plant (voucher number: YA-001) was authenticated and stored at the National Herbarium (Ethiopia), Addis Ababa University.

### 2.2. Methods

#### 2.2.1. Extraction of *Aloe* Mucilage

The yellowish mucilage obtained from the cut leaves was homogenized with water in a blender and strained to remove excess water. The gum was precipitated from the slake by soaking diethyl ether, and it was filtered by using suction filter, then spreaded, and allowed to dry in the air before oven-drying at 40°C for 4 h. The dried mucilage was pulverized, passed through a 224 *µ*m sieve, and stored in a desiccator in an airtight glass bottle for future use [16].

#### 2.2.2. Phytochemical Screening

The extract was screened for the presence of mucilage, and different phytochemical constituents including alkaloids, glycosides, terpenoids, tannins, and flavonoids using standard procedures were mentioned elsewhere [17, 18].

#### 2.2.3. Preparation of Zinc Oxide Suspension

Nine batches of the zinc oxide (ZnO) suspensions were formulated at 1%, 2%, and 4% *w/v* concentrations of the three suspending agents (Table 1): *Aloe weloensis* mucilage, acacia, and NaCMC. A 5 g quantity of ZnO powder was first levigated with glycerin (1 : 1, *w/w*). 1 g of aloe mucilage was triturated together in a dry porcelain mortar until properly mixed, and then 0.05 g of preservative (methylparaben) was added and further triturated. Distilled water was added to form a pourable paste which was transferred to the 100 mL measuring cylinder and made up to 100mL volume with water and shaken vigorously for 3 min. The procedure was repeated using 2% *w/v* and 4% *w/v* of the *Aloe weloensis* mucilage, acacia gum, and NaCMC [16, 19].

#### 2.2.4. Evaluation of Suspensions

*(1) pH Measurements*. The pH of all the prepared ZnO suspensions was measured weekly for four weeks using digital pH meter (Hanna Instruments, HI 8314, Singapore) [20]. The pH meter was calibrated before use with a standard solution of known pH at pH 4.01 and 7 at room temperature.

*(2) Sedimentation Volume*. Sedimentation volume was determined by the following procedure mentioned elsewhere [1]: the formulation (20 mL) was poured into a 25 mL measuring cylinder and kept at room temperature. The sedimentation volumes (%) of the formulations were observed daily for the first 7 days after which the observations were performed weekly for 4 weeks. The readings of the sedimentation volumes (%) were taken where the clear supernatant starts to become cloudy upon descending from the top surface of the suspension. The rate of sedimentation (*s*^*r*^) was calculated using the equation as follows [2]:

(1) $$\begin{array}{c} s_{r}=\frac{H_{t}}{H_{o}}*100, \end{array}$$

where *H^t^* is the ultimate volume of the sediment and *H^o^* is the original volume of the suspension.

#### 2.2.5. Measurement of Viscosity

The viscosity of the suspensions was determined at 25^o^C using the Brookfield viscometer model-RVDV PRO II (Brookfield, USA) at 100rpm (spindle number 4) within 48 hours of preparation. All determinations were made in triplicate, and the results obtained were expressed as the mean values [19].

#### 2.2.6. Determination of Flow Rate

The time required for 10 mL of suspension to flow through an orifice of 10 mL pipette was determined. The average of three readings was consistently recorded, and the flow rate was calculated by using the following equation:

(2) $$\begin{array}{c} Flow\;rate=\frac{v^{s}}{T}, \end{array}$$

where V^s^ is volume of sample in the pipette (in mL) and *T* is the time (in sec) required for the 10 mL suspension to elute out of the pipette [20].

#### 2.2.7. Redispersibility

The redispersibility was evaluated according to a methods described elsewhere [19, 21]. 20 mL of the suspension was poured into a 25 mL measuring cylinder and allowed to stand for a week. The measuring cylinders were manually and gently rotated at 180^o^. The formulations were evaluated based on the number of turns (one complete cycle) required to uniformly redisperse the sedimented particles throughout the suspension.

#### 2.2.8. Statistical Analysis

All data reported in this study were the averages of triplicate determinations. Wherever appropriate, the data were subjected to statistical analysis using Origin software version 7.0 (OriginLab Corporation, MA, USA). In all cases, individual differences and all other relevant data were evaluated using Tukey's test for a one-way analysis of variance (ANOVA). *p* value of less than 0.05 was considered to be significant.

## 3. Results and Discussion

### 3.1. Phytochemical Screening of the Mucilage

The mucilage from *Aloe weloensis* appeared light yellowish and was found to swell in contact with water, giving a highly viscous solution. Preliminary phytochemical screening of the sample showed the presence of glycosides, mucilage, tannins, and flavonoids except alkaloids. The preliminary screening result is presented in Table 2.

### 3.2. Evaluation of Suspensions

#### 3.2.1. Sedimentation Volume

The performance of natural gums and mucilage is evaluated based on suspending ability and stability of the suspension, which is assessed in pharmaceutical formulations [22]. The rate of sedimentation of the suspending agent provides an idea about the suitability of the suspending agent in pharmaceutical formulations. The suspending agent is considered better if its sedimentation rate is less [16].

The sedimentation volumes (%) of the suspending agents used in this study are presented in Table 3. As it can be seen from the table, all concentrations of *Aloe weloensis* mucilage exhibited high sedimentation volume which is an indicative of better suspending characteristics which were comparable with acacia and NaCMC. On the first day, all suspensions showed similar sedimentation volume 100%. During the first few days, *Aloe weloensis* mucilage showed comparable sedimentation volume with both NaCMC and acacia. However, as time of storage increases, the sedimentation volume of aloe mucilage suspension showed a significant reduction as compared to NaCMC which might be attributed to easy degradation of natural polymers over a prolonged period that the structured vehicle will be disrupted which makes the suspending agents unable to maintain the particles suspended. At 1% and 4%, both *Aloe weloensis* and acacia showed comparable sedimentation volume (*p* > 0.05). The study result also showed that there is direct proportionality between mucilage concentration and sedimentation volume as indicated in Table 3. There is an increase in the sedimentation volume when the concentration was increased from 1% to 2% and to 4% (optimum concentration). Previous studies have suggested *Abelmoschu*s *esculentus* and *Hibiscus lobatus* leaves to be better suspending agents compared to traditional suspending agents such as tragacanth and NaCMC [23, 24] which is in line with our study findings.

#### 3.2.2. Viscosity and Flow Rate

Suspensions are the least stable dosage form due to sedimentation and cake formation. As the viscosity of the suspension increases, the terminal settling velocity decreases; thus, the dispersed phase settles at a slower rate and remains dispersed for a longer time yielding higher stability to the formulated suspension. The less viscous suspension tends to pour more easily than the more viscous ones, and hence, the study of viscosity is critical to understand the stability of suspensions [25].

**Figure 1:**
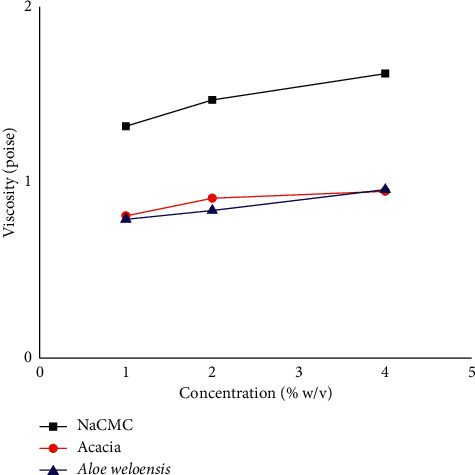
Comparative viscosities of ZnO suspensions prepared using different concentrations of *Aloe weloensis* mucilage, acacia gum, and NaCMC as suspending agent.

In this study, viscosity of the suspensions was studied and the results are tabulated in Table 4. The results indicated that as the amount of the suspending agent increases, viscosity also increases gradually. Suspension prepared using *Aloe weloensis* mucilage and acacia showed comparable viscosity (*p* > 0.05). However, the suspension from NaCMC was more viscous than the other two suspensions (*p* < 0.05). The viscosity of ZnO suspensions prepared from the three suspending agents is shown in Figure 1. The suspension containing NaCMC was found to be most viscous; the ranking was NaCMC > acacia > *Aloe weloensis* at 1% and 2%. On the other hand, at 4%, *Aloe weloensis* mucilage had a higher viscosity (0.96 poise) than acacia (0.91 poise) (Table 4).

The flow rate of the suspension was inversely proportional to the concentration of the mucilage. Even high concentrations of NaCMC showed a very low flow rate due to its highly viscous nature. It was not possible to determine the exact flow rate of NaCMC through 10 ml pipette at higher concentrations (4%) because of highly viscous nature. The flowability of the suspensions, at all concentration levels of the suspending agents, were in the order of acacia > *Aloe weloensis* mucilage > NaCMC which may be attributed to the difference in viscosity. The increase in viscosity will lead to the decrease in flowability (Table 3).

#### 3.2.3. pH Measurement

The pH of the suspension indicates the acidic or basic nature of suspensions. Change in pH should be considered when suspensions are prepared with drugs. The pH of the suspensions prepared ranges from 7.1 to 8.6. Slightly basic to near-neutral pH of *Aloe weloensis* suspensions (7.4 to 8.3) showed their suitability. The pH values of the zinc oxide suspensions formulated at 1%, 2%, and 4% w/v concentrations of NaCMC, acacia, and *Aloe weloensis* used as suspending agents as shown in Table 5 indicated that the three suspending agents were fairly stable on storage for 4 weeks. Thus, the study revealed that the extracted mucilage of *Aloe weloensis* may be used as potential pharmaceutical adjuvants even at low concentrations (1, 2, and 4% *w/v*) concerning pH.

#### 3.2.4. Redispersibility

Suspensions must be readily dispersible to ensure uniform dosage administration of the medicament after shaking. If the sediment remains even after shaking vigorously for a specified time, the system is described as caked [26]. The redispersibility of all formulated suspensions was studied and compared with each other. All these suspensions were found to be easily redispersible after maximum turns required to rotate 180˚after storage for 45 days (Table 5). However, to redisperse NaCMC suspensions, more shaking time was required as compared to the other suspensions formulated with acacia and *Aloe weloensis*. The redispersibility of the suspensions with a lower concentration of suspending agents (lower viscosity) was quicker than that of suspensions formulated with higher concentration of suspending agents (higher viscosity). This may be attributed to the higher viscosity of the formulations with higher concentration of the suspending agents. The redispersion ability of the suspending agents was found to be uniform for the entire suspending agent.

## 4. Conclusion

*Aloe weloenesis* has good suspending qualities which are comparable with the standard materials used for this work. The sedimentation volumes (%) and ease of redispersibility of the suspensions were in the order of NaCMC > *Aloe weloensis* mucilage > acacia. Thus, it can be concluded that the extracted mucilage from the leaves of *Aloe weloensis* has the potential to be used as a suspending agent. But the stability and compatibility (which were not performed due to machine constraints in our setup) of the formulation in pilot scale need to be emphasized in the future.

## Figures and Tables

**Table 1:** Formulation ingredients of the zinc oxide suspensions.

Formulation ingredients	Composition (% w/v)
Zinc oxide	5
Suspending agent^*∗*^	1, 2, and 4
Methylparaben	0.05
Glycerin	1
Distilled water qs^*∗∗*^	100 ml

^*∗*^The suspending agents used are mucilage of *Aloe weloensis*, acacia and, NaCMC each at the specified concentration. ^*∗∗*^Quantity sufficient.

**Table 2:** Preliminary phytochemical screening of *Aloe weloensis* mucilage.

Tests	Observations
Test for tannins (ferric chloride test)	Positive
Test for flavonoids (Shinoda test)	Positive
Test for mucilage (ruthenium red test)	Positive
Test for alkaloids (Wagner's test)	Negative
Test for glycosides (Keller Killiani)	Positive

**Table 3:** Sedimentation volume of ZnO suspensions prepared from *Aloe weloensis*, acacia, and NaCMC at different concentrations.

Suspending agent	Concentration (% w/v)	Sedimentation volume									
		Time (days)							Time (weeks)		
		1	2	3	4	5	6	7	2	3	4
NaCMC	1	1	0.95	0.95	0.93	0.93	0.93	0.93	0.9	0.86	0.8
	2	1	0.98	0.98	0.96	0.95	0.93	0.93	0.93	0.93	0.93
	4	1	1	0.983	0.98	0.98	0.96	0.96	0.96	0.96	0.96

Acacia	1	1	0.83	0.83	0.8	0.8	0.76	0.73	0.56	0.33	0.23
	2	1	0.9	0.9	0.9	0.9	0.9	0.9	0.8	0.73	0.7
	4	1	0.9	0.9	0.93	0.9	0.9	0.9	0.9	0.5	0.46

*Aloe weloensis mucilage*	1	1	0.9	0.83	0.8	0.76	0.66	0.66	0.46	0.43	0.4
	2	1	0.93	0.86	0.83	0.76	0.73	0.5	0.5	0.48	0.43
	4	1	0.96	0.9	0.86	0.8	0.76	0.67	0.5	0.53	0.51

**Table 4:** Viscosity (poise) and flow rates (mL/sec) of ZnO suspensions prepared at different concentrations of *Aloe weloensis* mucilage, acacia, and NaCMC at room temperature.

Suspending agent	Concentration (%, *w/v)*	Flow rate	Viscosity
NaCMC	1	0.18	1.32
	2	0.12	1.47
	4	—	1.62

Acacia	1	1.25	0.81
	2	0.66	0.91
	4	0.4	0.95

*Aloe weloensis mucilage*	1	0.66	0.79
	2	0.4	0.84
	4	0.2	0.96

^—^The suspension prepared from this suspending agent is too viscous that it cannot flow from the pipette.

**Table 5:** Rate of redispersibility and pH of ZnO suspensions formulated from the three suspending agents.

Suspending agents	Concentration (% *w/v)*	pH				Rate of redispersibility (cycle)
		Storage duration (week)				
		0	1	2	3	After 45 days
NaCMC	1	7.6	8.2	8.5	8.7	10
	2	7.7	8.3	8.6	8.6	15
	4	7.7	8.0	8.5	8.5	17

Acacia	1	7.2	7.8	8.0	8.0	7
	2	7.1	7.7	7.9	8.0	9
	4	7.3	7.9	8.2	8.2	14

*Aloe weloensis* mucilage	1	7.5	7.7	7.9	8.2	6
	2	7.8	8.0	8.0	8.3	7
	4	7.4	7.9	7.9	8.0	9

## Data Availability

The datasets used and/or analyzed during the current study are available from the corresponding author on reasonable request.

## Abbreviations

NaCMC: Sodium carboxy methylcellulose
ZNO: Zinc oxide
RPM: Revolutions per minute
